# Clause Chaining and Discourse Continuity in Turkish Children's Narratives

**DOI:** 10.3389/fpsyg.2020.00115

**Published:** 2020-02-19

**Authors:** Hale Ögel-Balaban, Ayhan Aksu-Koç

**Affiliations:** ^1^Psychology Department, Bahçeşehir University, Istanbul, Turkey; ^2^Psychology Department, Boğaziçi University, Istanbul, Turkey

**Keywords:** clause chaining, clause combining, Turkish, narrative, children

## Abstract

The present study examines the development of complex sentences with non-finite clause combining with particular focus on clause chaining, in narratives of 40 Turkish-speaking 4- to 11-year-olds and six adults elicited by a wordless picture book. Results show a gradual increase by age in the variety of clauses combined, the length of the complex sentences and their frequency of use. Clause chains formed with converbal clauses are the earliest and most frequent type of clause combinations, already present in 4-year-olds' complex sentences with 1-non-finite clause. Older children's and adults' 2- or 3-non-finite clause complex sentences consist of some combinations of adverbial, complement, relative and converbal clauses. Developmentally, clause chains establish first, aspectual-temporal continuity, then temporal-causal continuity. Sentence-internal and cross-sentence-boundary referential continuities are present early, from age 4 onwards. These findings are discussed in terms of the demands of narrative organization as well as the syntactic and semantic complexity of the clause combination devices in Turkish.

## Introduction

Discourse, conversational or narrative, revolves around one or more topics. In conversation, speakers and listeners co-construct a topic in order to achieve a coherent account of what is at issue (Ervin-Tripp and Küntay, 1997). However, in narratives it is the task of the speaker to create coherence by organizing events in accordance with a goal motivated by the cognitive and affective states of the actors engaged (Labov and Waletzky, 1967; Bruner, 1990). As Givón (2017a, p. 29) puts it, “… coherent discourse tends to maintain, over a span of several clauses, the same topical referent, the same or contiguous time, the same or contiguous location, and sequential action.” Thus, among the primary skills that children have to acquire in order to become effective conversationalists or narrators are linking the sequence of events temporally and causally and making clear reference to actors so that the listener can follow who is doing what to whom. That is, to effect connectivity in discourse, children have to master the clause combining and referent-tracking devices of their language.

Clause combining involves “the combination of a clause with some other constituent” (Gast and Diessel, 2012, p. 3). The two types of combination traditionally recognized are coordination and subordination. In coordination, “[at least] two constituents belonging to the same category are conjoined to form another constituent of that category” (Kroeger, 2005, p. 218), while in subordination one of the constituents is embedded and also dependent on the other for tense and person marking. A third type of linkage is one where the syntactic combination is neither perfectly symmetrical as in coordination, nor does it involve embedding as in subordination (Sarvasy and Choi, forthcoming) but the sequenced clauses are dependent on the main clause. This type of linkage is characteristic of clause chaining which involves the sequencing of one or more clauses with verbal predicates that are under-specified for tense (and, often, other categories) combined with a single clause of which the predicate bears full tense, mood and subject reference marking (Sarvasy and Choi, forthcoming). The clauses in a chain are thus syntactically dependent but not embedded. The non-finite verbal predicates may indicate aspectual distinctions such as perfectivity and imperfectivity, temporal relations such as sequence and simultaneity as well as relations of cause-effect (Longacre, 2007, p. 400). The morphology of the non-finite verb forms may also indicate whether the subject of each should be interpreted as the same (SS “same subject”) or different (DS “different subject”) from the subject of the preceding or the following clause (Stirling, 2006; Longacre, 2007, p. 375).

Considering the relation between clause structure and referential mechanisms, Givón (2017a, p. 25) notes that “there is a correlation between referential continuity and non-finiteness” such that grammatical devices that are most finite indicate the least degree of anaphoric referential continuity, while those that are least finite mark the highest referential continuity. In an analysis of clause chaining in Japanese, Watanabe (1994, p. 141) argues that chaining devices mark “action/event continuity [that] has to do with the predictability or conceptual connectedness of action/event sequences.” Two events are said to be conceptually more tightly connected when there is a larger degree of information overlap between them, which tends to be in terms of the most recurrent information in grammar, that is, referent, time, location and tense-aspect-mood (Watanabe, 1994, p. 142; Givón, 2017a). For example, two simultaneously occurring events are more likely to be perceived as a unit if the same actors (subjects) are involved, but may not be perceived so if the actors are different. Thus, it is through various combinations of their aspectual-temporal, locational and referential properties and the semantics of the verb, that chaining devices, create action/event continuity in discourse (Watanabe, 1994, p. 142).

Here, we target an analysis of the developmental trajectory of clause chaining in Turkish, a zero anaphora language. Although our focus is on (i) chains with converbs that involve dependency but not embedding (henceforth “clause chaining”), we also include in our analysis (ii) combinations with complement, relative and adverbial clauses that are both dependent and embedded (henceforth “clause combining”). For this purpose, we analyze narrative data from children and adults and trace changes in clause chaining and combining, asking whether the development of the two types of complex sentences proceeds at different paces. We also ask whether there is a change with age in the role the aspectual-temporal and referential properties of chaining devices play in discourse continuity.

The organization of the paper is as follows. We first briefly describe the reference tracking and clause linkage devices of Turkish. Then, we refer to previous studies on clause chaining and combining. After presenting our methodology and results, we offer a discussion of the findings.

## Reference Marking and Non-finite Clause Chaining and Clause Combining in Turkish

### Reference Marking

Turkish is an agglutinating language with flexible SOV order which is subject to pragmatic constraints. In the canonical order, non-finite clauses precede the main clause. There is no formal article system; indefiniteness is marked by the numeral *bir* “one,” definiteness by the accusative case and by bare nouns. There is no grammatical marking of gender or animacy either in the nominal or the pronominal systems, and the third person singular pronoun *o* “he/she/it” has the same form as the demonstrative pronoun *o* “that.” Clauses may lack overt subjects since the verb is marked by a person suffix for agreement. The use of pronouns, overt or null, is determined by the discourse context, and conveys pragmatic information such as contrast, similarity, emphasis or new information (Enç, 1986; Erguvanlı Taylan, 1986; Öztürk, 2001; Göksel and Kerslake, 2005; Azar et al., 2016). Once the discourse topic is set with a noun phrase or overt pronoun, continuity requires the use of null pronouns. But when the speaker wants to mark topic change, an overt pronoun is necessary for grammaticality (Öztürk, 2001, p. 240)

In short, Turkish uses overt or null pronouns as anaphoric expressions representing coreferentiality with another noun phrase (NP). To summarize from Erguvanlı Taylan (1986, p. 214–215, 217, 223), in simple sentences, coreference with a subject NP is expressed by zero anaphora regardless of the position of the subject. In embedded sentences, the subject is expressed with a zero representation when it is to be interpreted as coreferential with the main clause subject. In discourse, anaphoric relations extend beyond the boundaries of the sentence and the antecedent may be in the discourse context the sentence occurs in. Thus, in Turkish, it is null pronouns that go hand-in-hand with discourse continuity rather than pronouns.

### Non-finite Clause Types Functional in Clause Chaining and Clause Combining

Four types of non-finite clauses stand out as participants in clause chaining and clause combining in Turkish. Ordered in terms of a cline of dependency and embedding, these are relative and complement clauses, adverbial clauses and converbal clauses. Among these, only converbal constructions are functional in clause chaining.

Turkish makes use of participial suffixes (-*En*, -*DIK, -EcEk, -mIş and -Ir)* to form relative clauses, and nominalizing suffixes (-*DIK, -EcEk, -mEk, -mE*) to form complement and adverbial clauses.^1^ In all three construction types, one of the respective suffixes is attached to the verb stem, which is followed by the possessive suffix for person/number and case in relative and complement clauses, and by case and/or a postposition in adverbial clauses (Erguvanlı Taylan, 2015, p. 207). The participial or nominalizing suffix occupies the same position as tense markers on the finite verb but may carry only aspectual-temporal or modal values depending on the semantics of the non-finite verb and the main verb. Since they have their own agreement markers, the nominal/pronominal subjects of these constructions can be omitted just as in main clauses (Kornfilt, 1997, p. 45–46, 48). In relative and complement clauses, the overt subject, if present, is marked with the genitive case. In complement clauses, the choice of the nominalizing suffix to be used is determined by the semantics of the main verb (e.g., *-DIK* with verbs of cognition). With this requirement and that for genitive marking on overt subjects, complement clauses pose some syntactic and semantic complexity for acquisition (Aksu-Koç, 1994). In adverbial clauses, simpler in both respects, the nature of the relationship between the two clauses (e.g., temporal or causal) is expressed either by a case marker or a postposition. Example (1) illustrates a complex sentence with an adverbial and a complement clause^2^.


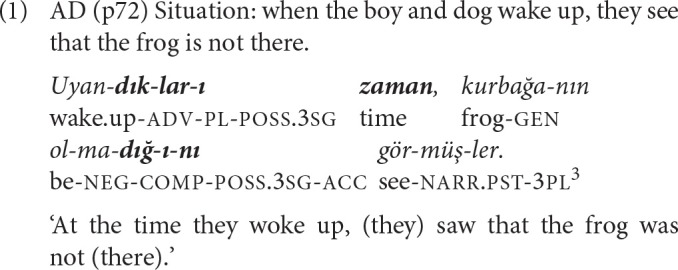


Converbs, also adverbial in function (Kornfilt, 1997; Göksel and Kerslake, 2005), are derived from verbs by attaching a morphologically unanalyzable suffix (Johanson, 1995, p. 315) or have a composite structure formed by a nominal suffix followed by case or a postposition. Göksel and Kerslake (2005, p. 404) note that the “most important structural distinction among converbs is between those that are marked for person and those that are not.” In the present study, only those converbs that are not marked for person have been included in the analysis of chaining devices. Some converbs encode coreferentiality between the subjects of the clauses they join (SS), whereas in others, called “variable subject” converbs by Haspelmath (1995, p. 10) or “open converbs” by Johanson (1995, p. 318), the subjects of the two clauses may be the same or different (SS/DS). Among the converbs that appear most frequently in our data are those formed with the suffixes -*Ip*, -*ErEk, -IncE, -ken*, and *-DIktEn sonra*.

Converbs with *-Ip* and *-ErEk* suffixes join clauses with shared arguments, hence they are SS devices which indicate that the subject of the upcoming clause is the same as the present one (Givón, 2017b, p. 106). Converbs derived with *-IncE, -ken* and *-DIktEn sonra* may connect clauses with shared or different arguments. In SS uses, the coreferentiality of the subject of the two clauses is expressed with a null pronoun. If the subject of either the main or the adjunct clause is represented by a pronoun or an NP, the converbal construction is interpreted as DS (Erguvanlı Taylan, 1986, p. 216).

The suffix *-Ip* corresponds to the general conjunction “and/and then” and joins clauses “that are semantically of equal status in the sentence” (Göksel and Kerslake, 2005, p. 410)^4^. Aspectually, -*Ip* marks perfectivity and a sequential relation with the event of the following clause if the predicates of both clauses are action verbs, and an almost single event interpretation if one of the predicates is a verb of cognition. The suffix *-ken* “while doing/being” is added to verbs already inflected for aspect, most often with the aorist *-I/Ar* that denotes imperfectivity. *-ken* expresses simultaneity with full or partial overlap between two events, depending on whether the verb it is attached to is perfective or imperfective. In example (2), an *-Ip* and a *-ken* clause form a SS chain that presents the action (looking) and the perceptual experience (seeing) of the same actor as the context for the perfective event performed by a different actor, the subject of the main clause.


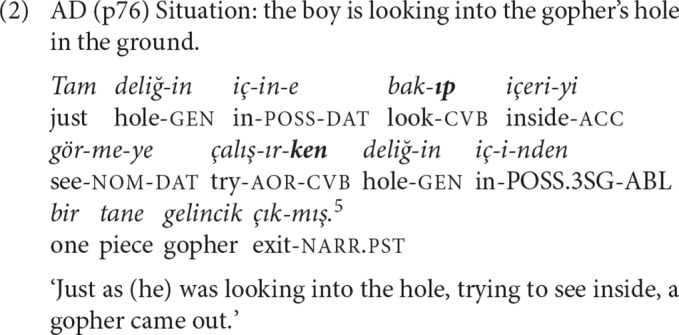


Converbs derived with the suffix *-IncE* indicate perfectivity, the completion of the event expressed by the *-IncE* clause, resulting in the inception of the event expressed in the following clause. *-IncE* expresses a sequential temporal relation, meaning ‘when/cause' or ‘as soon as.' Example (3) is a complex sentence where an *-IncE* clause is followed by an adverbial clause with the same subject, both connected to the main clause that has a different subject.


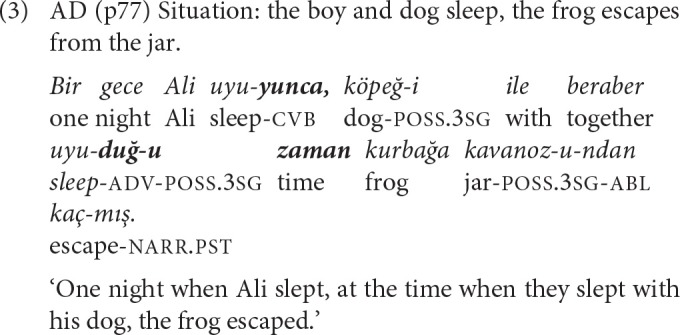


*-ErEk* ‘by doing/in doing' functions both as a conjunction and an adverb describing manner of action (Göksel and Kerslake, 2005). Aspectually, *-ErEk* clauses get an imperfective interpretation. In a comprehensive analysis of the functions of Turkish converbs, Slobin (1995) observes that the event in the *-ErEk* clause is presented either as a preparatory, or an instrumental, or a simultaneous accompanying phase for the situation mentioned in the main clause. *-ErEk* thus integrates two situations as different but co-existential aspects of a single activity. The complex sentence in (4) describes the subject with a relative clause and the manner of his action with an *-ErEk* clause.


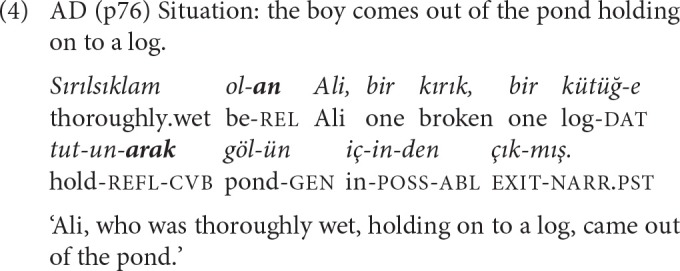


The SS/DS *-DIktEn sonra* ‘after verb-ing' is a composite structure formed by appending the nominalizing suffix *-DIK* and the ablative case *-DEn* to the verb stem, followed by the postposition *sonra* ‘after' (Göksel and Kerslake, 2005, p. 429). *-DIktEn sonra* clauses indicate perfective aspect and an ordered temporal relation. Example (5) is an SS chain formed with the converbs *-DIktEn sonra*, and *-Ip*, and a relative clause modifying the object of the *-Ip* clause.


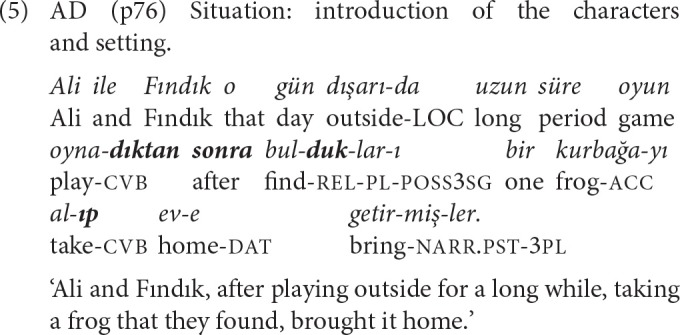


The semantic interpretation of converbs differs in terms of their specifity vs. context dependence. Slobin (1995, p. 356) draws an important distinction between *-IncE* and *-ken* vs. *-Ip* and -*ErEk*, in that the first two are inherently temporal whereas the last two rely on contextual inferences for their interpretation as successive or simultaneous. *-DIktEn sonra* also explicitly expresses a temporal relation. All five converbs contribute to aspectual-temporal and referential continuity between clauses in chained sequences.

The above examples from the adult data show that Turkish converbs, in addition to connecting sequences of events in chains, also combine with complement, relative or other adverbial clauses in complex sentences.

## Research on the Development of Connectivity in Turkish

Crosslinguistic evidence on connectivity has demonstrated that children acquire coordinate constructions by age 3 (Bloom et al., 1980; Peterson and McCabe, 1988) and complex sentences with subordination by age 4 (Diesel and Tomasello, 2001; Givón, 2001), showing further developments until 7–12 years of age (e.g., Berman and Slobin, 1994a: English, German, Hebrew, Spanish, Turkish; Justice et al., 2006: English; Kit-Sum To et al., 2010: Cantonese; Mäkinen et al., 2014: Finnish; among others).

Research on Turkish similarly indicates that conjunctions, converbs, adverbial, relative, and complement clauses are acquired between ages 2;6 to 5;0 but the flexibility of use in narrative discourse continues to develop until early school years (Slobin, 1986, 1995; Aksu-Koç, 1994; Aksu-Koç and von Stutterheim, 1994; Küntay and Nakamura, 2004; Altan, 2008; Özge et al., 2010; Nakipoğlu and Yıldız, 2014; Sarılar et al., 2015). Studies on the development of referent tracking also evidence gradual progress during the preschool and school years (Özcaliskan and Slobin, 1999; Küntay, 2002; Aksu-Koç and Nicolopoulou, 2015).

Although there are no studies that specifically focus on input concerning connectives, children's own use in spontaneous conversations as well as in narratives indicate that they hear these forms early in development. Slobin provides evidence for their frequent use in books for preschool children (1995, p. 350) and from cros-sectional data of adult-child conversation where children's uses of converbs *-IncE* and *-ken* were recorded at 2;0 and of *-Ip* and -*ErEk* at 3;0 (1982, cited in 1995, p. 350). In this same data set, Altan (2005) observed that although children produce nominalized complements around 3;0, they prefer using sentential complements. In the longitudinal data of two children between 1;6 and 3;3, complement clauses were infrequent in the input either because mothers simplify their speech by using sentential complements or because there are few occasions to use them (Altan, 2005).

The development of clause linking in narrative discourse has been described for English, Hebrew, German, Spanish and Turkish in the now-classic work by Berman and Slobin (1994b) and for many other languages in studies in the same tradition. This body of research on stories elicited by use of the picture book *Frog where are you?* (Mayer, 1969) has revealed that younger children use coordinating and older children subordinating constructions to package several related events into syntactically larger units or chunks by early school years (Aksu-Koç, 1994; Aksu-Koç and von Stutterheim, 1994; Bamberg, 1994; Berman and Slobin, 1994a; Slobin, 1995; Aarsen, 1996; Fernández, 2013; among others).

Berman and Slobin (1994a, p. 540–541) summarized the patterns observed crosslinguistically across the original five languages and observed that Turkish children, in line with the typological pattern of their language, prefer non-finite linking with converbal and infinitival constructions instead of connecting finite clauses with subordinating or non-subordinating conjunctions that are favored by peers speaking the other languages. Further evidence for Turkish children's early usage of converbal clauses in narratives and conversation is provided by Çapan (2013) and Rehbein and Herkenrath (2015). Both studies report an increase in the use of converbs between ages 4 and 5 in terms of frequency, variety and function. Converbs *-ken*, -*IncE*, and -*Ip* were scarce and limited to expression of temporality in the speech of 4-year-olds, whereas in the speech of 5-year-olds the frequency of their use increased and was not functionally restricted, expressing causality, manner and temporality. The speech of 5-year-olds and older children also displayed the use of other converbs such as *-ErEk*.

In the present analysis, we build on this previous work (Aksu-Koç, 1994; Berman and Slobin, 1994a,b; Çapan, 2013; Rehbein and Herkenrath, 2015) by tracing the use of chains and combinations of non-finite clause structures in complex sentences. Our research questions ask the following: (1) What is the developmental trajectory of complex sentences with clause chains and clause combinations as revealed by their frequency of use and the number and types of clauses combined? (2) What patterns of discourse continuity in clause chains—aspectual-temporal and referential—can be identified across age groups?

## Method

### Participants

Forty native Turkish-speaking children and 6 adults participated in the study. The distribution of the participants by age and sex is as follows: 10 4-year-olds (6 males, *M*_*age*_= 4;8, range = 4;1–4;11), 10 5-year-olds (4 males, *M*_*age*_ = 5;6, range = 5;0–5;10), 10 8-year-olds (3 males, *M*_*age*_ = 7;8, range = 7;7–8;3), 10 11-year-olds (6 males, *M*_*age*_ = 11;2, range = 11;0–11;11) and 6 adults (1 male, *M*_*age*_ = 21;0, range = 20;7–22;0). Four- and 5-year-old participants were recruited from kindergartens and 8- and 11-year-olds from primary and secondary public schools in Istanbul, Turkey. The adults were psychology undergraduates at Istanbul Bilgi University in Istanbul, Turkey. All participants were of middle socioeconomic background. Appropriate permissions were obtained for their participation.

### Material and Procedure

#### Narrative Production Task

Narratives were elicited using Mayer's wordless picture book ‘*Frog, where are you?*' (1969). The story depicted is about a boy, his dog and his lost frog. In their search for the frog, the boy and the dog go through successive encounters with different animals (a gopher, an owl, bees, and a deer) in the woods and finally find their frog and take it back home. The events of the story, represented in 24 pictures, are conducive to clause chaining and combining (Ögel-Balaban, 2015).

#### Procedure

Each participant was tested individually in a separate room in their schools. The experimenter introduced the book and asked the participants to first look through all the pictures and then tell the story. She did not provide any prompts during story telling. Both the participant and the experimenter had the pictures of the book open in front of them as the story was being told. The narratives were video-recorded.

### Transcription and Coding

Video-recordings of the narratives were transcribed using EUDICO Linguistic Annotator (ELAN), developed at the MPI for Psycholinguistics, Nijmegen, for the analysis of language, sign language and gestures (Lausberg and Sloetjes, 2009). The data analyzed consist of a total of 1,833 clauses (6,485 s) comprising the narratives of 4- to 11-year olds and a total of 373 clauses (1,143 s) comprising the narratives of adults.

The narratives were coded clause by clause. A clause was defined as a unit minimally consisting of a pairing of a predicate and a set of arguments (Gast and Diessel, 2012, p. 3). Each clause was coded for the following parameters:

(i) Type of sentence in which the clause occurs (code applies for each clause in the sentence):

Simple sentence: consists of a single clause with one verbal or nominal predicate marked for tense-aspect-mood and person/number.

Complex sentence: consists of a main clause with one verbal or nominal predicate marked for tense-aspect-mood and person/number and one or more embedded and/or dependent clauses.

(ii) Type of embedded and/or dependent clause:

Non-finite clauses that are dependent and embedded, and associated suffixes:

Adverbial (-*DIK*, -*EcEk, -mE);* complement (-*DIK*, -*EcEk, -mE)*, relative (-*En*, -*DIK*, -*EcEk, -mE)*^6^

Non-finite clauses that are dependent but not embedded, and associated suffixes:

Converbal (*-Ip, -ErEk, -IncE, -ken, -DIktEn sonra*)

Finite clauses that are embedded but not dependent on the main clause:^7^

Clauses conjoined by the conjunction *diye* ‘for' used in purpose clauses (e.g., *Kaç-ma-sın* ***diye****kavanoz-a koy-du-k* (escape-neg-imp.3sg for jar-dat put-pst-1pl) ‘We put it in the jar **so that** it won't escape.').

Clauses conjoined by ***ki***as a complementizer (e.g., *Bir de bak-mış-lar* ***ki*** *kurbağa yok*. (one too look-prf-3pl comp frog nonexist) ‘and they just look, the frog is not there.')

Direct speech reports: Clauses representing exact utterances of the story characters followed by the verb *de* ‘say' were not coded for their content and not counted as part of the clause combination, but the associated non-finite clause was coded. For example, *Çocuk köpeğe “sessiz ol” der-****ken****kurbağa-yı gör-müş*. (boy dog-dat quiet be say-cvb frog-acc see-narr.pst) ‘The boy saw the frog while saying “be quiet” to the dog' was coded as a 1-NCL with *-ken* converb.

(iii) Type of referentiality for each clause type: Same Subject (SS), Different Subject (DS)

(iv) Number of clauses in a complex sentence with non-finite clauses:

1-NCL complex sentence: a non-finite clause and a main clause;

2-NCL complex sentence: two non-finite clauses and a main clause;

3-NCL complex sentence: three non-finite clauses and a main clause (includes the only two instances of 4-NCL combinations);

(v) Complex sentences with a syntactic or semantic error were included in the counts. They are displayed in **Table 6** and discussed in the text.

(vi) Subject markers: Each of the three characters, boy, dog and frog and all other characters combined as ‘others' were coded for the following subject marking devices, only when they were in the subject role: NP, PRO, POSSESSIVE on the verb, NULL PRO.

(vii) Aspectual-temporal continuity in clause chains and combinations:

Same aspect: the non-finite clauses express the same aspectual distinctions (e.g., perfective–perfective).

Different aspect: the non-finite clauses express different aspectual distinctions (e.g., perfective–imperfective).

Aspect and modality: At least one of the non-finite clauses expresses aspect, and at least one, modality.

An inspection of the narratives which contained complex sentences with 2- and 3-NCL chains and clause combinations showed that they all maintained an anchor tense, providing continuity at the discourse level (either the *-mIş* inflection as the narrative past or the *-Iyor* inflection as the narrative present). Aspectual-temporal characteristics of the chains and clause combinations which concern relations between events at the sentence level were coded as indicating ‘Same Aspect,' ‘Different Aspect' or ‘Aspect & Modality' on the basis of the aspectual meaning of the chaining and combining devices and the verbs they are attached to, as well as the aspectual meaning of the main verb. The tense-aspect marking on the main verbs, i.e., the anchor tense, which is the same across (almost) all utterances of a narrative, was not taken as criterial. For this reason, aspectual continuity was not traced in 1-NCL chains.

(viii) Cross-sentence boundary reference (XSR): Clauses were coded for XSR if the pronoun or null subject of a clause (non-finite or main) in a chain referred, for its identification, anaphorically to a referent expressed with an NP in a preceding clause external to the sentence, regardless of whether in subject or object role.

## Results

In view of the fact that the core element in clause chaining and clause combining is the verb and that there is a close relationship between lexical and syntactic development at younger ages (Marchman and Bates, 1994; Bastiaanse and Bol, 2001; among others), we used verb diversity as an index of general syntactic development. A one-way ANOVA on the mean number of different verbs by age revealed a significant difference between 4-year-olds and adults [*F*_(4, 41)_ = 4.18, *p* < 0.01]. Although 4-year-olds and older children did not differ significantly, the means show that starting at age 5 there is a gradual increase in the variety of verbs used by each age group (*M*_4-years_ = 20.80, *M*_5- years_ = 26.70, *M*_8- years_ = 29.40, *M*_11- years_ = 28.30, *M*_adults_ = 38.00) suggesting that the verb diversity of 4-year-olds is restricted as compared to that of older children, whose verb diversity, in turn, is not as extensive as that of adults.

A preliminary analysis using G^*^power (Faul et al., 2007) demonstrated that the sample size was too small for a powerful mixed-design inferential statistical analysis of the differences between age groups and their interactions with clause types. Therefore, no inferential statistical analysis was conducted regarding the data presented in the following sections.

### Overview of Syntactic Complexity of Narratives

Table 1 presents an overview of the syntactic complexity of children's and adults' narratives. First, the mean number of sentences shows an overall increase in the length of the narratives across age groups. Second, the relative percentages of simple sentences decrease (from 80% for 4-year-olds to around 50% for 11-year-olds and adults), and complex sentences increase (main and subordinate, from 20% for 4-year-olds to 45% for 11-year-olds and adults), indicating a change toward syntactic complexity by age.

**Table 1:** Mean number (and percentage) of total sentences, simple sentences, main, and subordinate clauses in complex sentences by age.

	**Total**	**Simple**	**Complex sentences**	
**Age**	**sentences**	**sentences**	**Main**	**Subordinate**
4-years	37.30	27.60 (79.79)	4.30 (9.16)	5.40 (11.05)
5- years	44.20	39.10 (65.79)	6.70 (15.22)	8.40 (18.99)
8- years	55.10	36.90 (66.50)	8.70 (16.08)	9.50 (17.43)
11- years	47.00	25.40 (55.47)	9.80 (20.42)	11.80 (24.10)
Adults	70.00	36.67 (50.56)	14.83 (21.78)	18.50 (27.65)

Table 2 displays the frequencies of three types of embedded and/or dependent clauses in complex sentences found in the data: Finite subordinate clauses including direct speech reports and clauses joined by conjunctions *ki* and *diye*, and non-finite subordinate clauses including those that are both embedded and dependent and those that are only dependent. Direct speech reports are used with high frequency by 4- and 5-year-olds, decrease in 8-year-olds' narratives and are rarely found in the narratives of older groups. Finite clauses linked with *ki* and *diye* are very few in 4- and 5-year-olds' narratives, are frequently used by 8-year-olds, but decline in the narratives of the older participants. Non-finite clauses that participate in complex sentences, on the other hand, show a steady increase across age groups (from 50% to 90%).

**Table 2:** Frequency (and percentage) of different types of embedded and/or dependent clauses by age^*^.

**Age**	**Total**	**Direct Speech**	**Finite**	**Non-finite**
4- years	54	26 (48.15)	1 (1.85)	27 (50.00)
5- years	84	20 (23.81)	5 (5.95)	59 (70.24)
8- years	95	11 (11.58)	18 (18.95)	66 (69.47)
11- years	119	3 (2.52)	4 (3.36)	112 (94.12)
adults	109	2 (1.83)	7 (6.42)	100 (91.74)

* *Non-finite = Converbs, adverbial clauses, complement clauses, and relative clauses; Finite = Conjunctions (diye, ki)*.

In the following analyses, direct speech reports and finite subordinate clauses are excluded, since our focus is only on clause chains and other non-finite clause combinations.

### Developmental Trajectory of Complex Sentences With Chains and Other Non-finite Clause Combinations

Our first research question asked about the developmental trajectory of complex sentences with chains and other non-finite clause combinations as revealed by their frequency of occurrence and the number and types of clauses constituting them.

Table 3 presents the frequency of complex sentences that have one (1-NCL), two (2-NCL) and three (3-NCL) non-finite clauses by age. It is observed that 100% of the 4-year-olds' complex sentences are composed of 1-NCL and a main clause, while 2-NCL complex sentences are produced by all older children. The narratives of 11-year-olds and adults have twice as many 2-NCL complex sentences as those of 5- and 8-year-olds. Examples of 3-NCL complex sentences are scarce overall.

**Table 3:** Frequency (and percentage) of complex sentences with 1-, 2- and 3-non-finite clauses (NCL) by age.

**Age**	**Total**	**1-NCL**	**2-NCL**	**3-NCL**
4- years	27	27 (100.00)	–	–
5- years	50	43 (86.00)	5 (10.00)	2 (4.00)
8- years	62	57	5	–
		(91.94)	(8.06)	
11- years	93	77 (82.80)	14 (15.05)	2 (2.15)
Adults	80	62 (77.50)	16 (20.00)	2 (2.50)

These data indicate that both the frequency of complex sentences and the number of clauses comprising them increase around age 5 but approximate those of adults sometime around age 11. The length of the complex sentences, however, do not exceed 3-NCLs in the present data (except for two cases of 4-NCLs by adults).

#### 1-NCL Complex Sentences

The frequency of 1-NCL complex sentences and of the associated clause types are presented in Table 4. The relative frequency of clause types is the same at successive ages, but both their frequency and variety increase. Converbal clauses, i.e., 1-NCL chains, are predominant at all ages, in particular for 4- and 5-year-olds, constituting about 85% of their complex sentences. Next in frequency are adverbial clauses, which show an increase at 8 years (33%), leveling off to 10–20% for the older groups. Complement and relative clauses are very infrequent in younger children's narratives but show a relative increase in the 11-year-olds' and adults' stories (on average 10% and 18%, respectively).

**Table 4:** Frequency (percentage) of types of non-finite clauses in 1-NCL complex sentences by age.

**Age**	**Total**	**CVB**	**ADV**	**COMP**	**REL**
4- years	27	23 (85.19)	3 (11.11)	1 (3.70)	–
5- years	43	37 (86.05)	4 (9.30)	2 (4.65)	–
8- years	57	34 (59.65)	19 (33.33)	4 (7.02)	–
11- years	77	53 (68.83)	8 (10.39)	10 (12.99)	6 (7.79)
adults	62	27 (43.55)	13 (20.97)	11 (17.74)	11 (17.74)

##### 1-NCL Chains

Table 5 presents the distribution by age of the five converbs that stand out as chaining devices in our narratives. At all ages, converbal clauses with *-Ip* have the highest relative frequency (40%), *-ken* (20–30%) and -*IncE* (10–30%) clauses have intermediate frequencies, and -*DIktEn sonra* (6–13%) and -*ErEk* (3–20%) clauses are at the lower end. The developmental order is, thus, *-Ip* > *-ken* and -*IncE* > -*DIktEn sonra* and -*ErEk*.

**Table 5:** Frequency (and percentage) of types of converbal clauses in 1-NCL complex sentences by age.

**Age**	**Total**	***-Ip***	***-ken***	***-IncE***	***-dIktEn sonra***	***-ErEk***
4- years	23	9 (39.13)	4 (17.29)	7 (30.43)	3 (13.04)	–
5- years	37	17 (45.95)	11 (29.73)	4 (10.81)	3 (8.11)	2 (5.41)
8- years	34	16 (47.06)	7 (20.59)	8 (23.53)	2 (5.88)	1 (2.94)
11- years	53	21 (39.62)	14 (26.42)	6 (11.32)	4 (7.55)	8 (15.09)
adults	27	10 (37.04)	7 (25.93)	2 (7.41)	2 (7.41)	6 (22.22)

As noted by Johanson (1995, p. 314–315), in chains constructed with SS suffixes, the verbs of the converbal and the main clauses may either form a common verbal phrase with no interposed elements between them, thus presenting the two events almost as a single event [example (6)], or there may be two full predicates presenting successive events [example (7)].


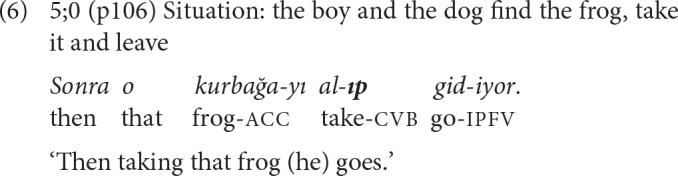


While children use *-Ip* early on, *-ErEk*, the other SS converb, is scarce in the narratives of the younger children, becoming more frequent in 11-year-olds' and adults' narratives. In example (7) from a 5-year-old, the *-ErEk* clause presents the first event as the instrumental/enabling phase for the second.


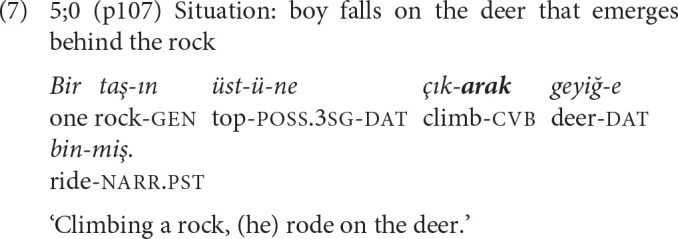


In DS chains, both the main and the converbal clause have a full verb as well as a subject of their own (Johanson, 1995, p. 314-315). Example (8) illustrates an 8-year-old's DS use of the simultaneity suffix *-ken* and example (9) an SS chain with *-IncE*.


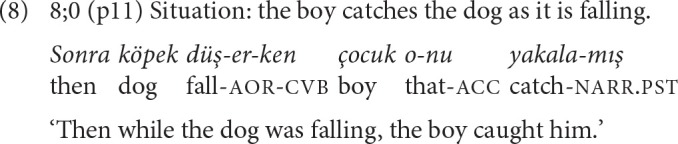



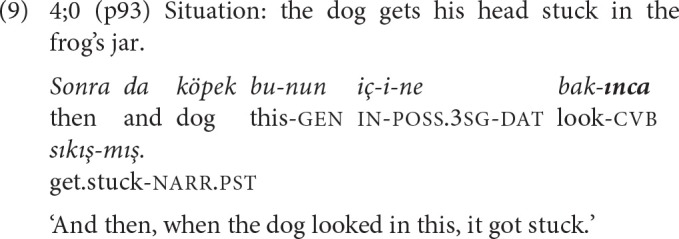


The overall frequency of *-ken* chains are higher than *-IncE* chains, and their DS uses are more frequent than SS uses most likely because the pictures of the story book depict the activities of the two protagonists simultaneously. The frequency of the SS vs. DS *-IncE* chains are relatively balanced.

Chains with the converb -*DIktEn sonra* are infrequent across age groups (around 10% or less). The SS chain in example (10) from an 8-year-old presents the events referred to by the two predicates in an explicit temporal relation.


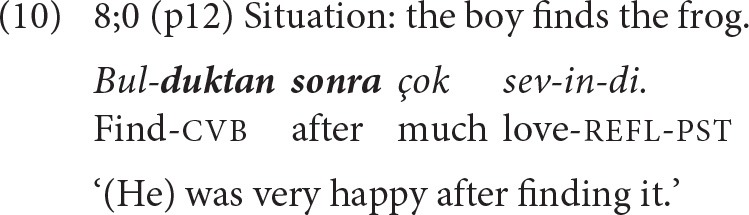


1-NCL chains with all of the five converbal suffixes are produced without error by 11-year-olds and adults. Although younger children use converbs early on and seem well aware of their structural requirements as noted by Çapan (2013, p. 109) (e.g., for an aspectual-temporal marker between the verb stem and the suffix -*ken*), they also make occasional errors. Table 6 presents the distribution of different types of errors observed in the data.

**Table 6:** Distribution of types of errors by age.

	**Syntactic error**						**Semantic error**	
	**Wrong chaining device**		**Missing genitive**		**Missing possessive**		**Semantically anomalous**	
**Age**	**1-NCL**	**2-NCL**	**1-NCL**	**2-NCL**	**1-NCL**	**2-NCL**	**1-NCL**	**2-NCL**
4- years	3	–	–	–	–	–	1	–
5- years	1	–	1	–	1	–	1	2
8- years	2	–	–	–	–	2	3	–
11- years	–	1	–	1	–	–	–	–
adults	–	–	–	–	–	–	–	–

One 5-year-old and two 8-year-olds use the SS suffix *-Ip* where the use of *-IncE* as a DS marker would be grammatical. In example (11), two clauses with different subjects are connected with *-Ip*, violating the requirement for the use of -*IncE*:


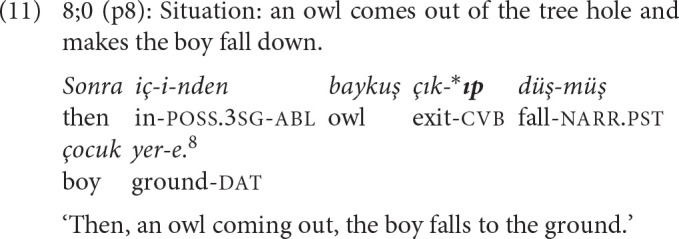


It has been noted that crosslinguistically, SS marking is the morphologically simpler means whereas DS marking may require an overt NP or an agreement marker (Stirling, 2006, p. 318). Although -*IncE* does not involve any agreement marking, it nevertheless appears to pose problems to children in its DS uses. The two forms may be confused because they both indicate perfective aspect and sequentiality, and *-Ip* may be accessed first because it is the earliest acquired and most frequently used form.

##### Other 1-NCL Combinations

As presented in Table 4 and noted above, the proportion of adverbial, complement and relative clauses in 1-NCL sentences is much lower than that of converbal clauses, particularly for 4- and 5-year-olds. These clause types that are both embedded and dependent are also morphosyntactically more complex than converbal clauses, hence their gradual increase across age groups.

Adverbial clauses are mainly used to express causal relations in the present narratives [example (12)], and complements refer to mental sates [example (13)].


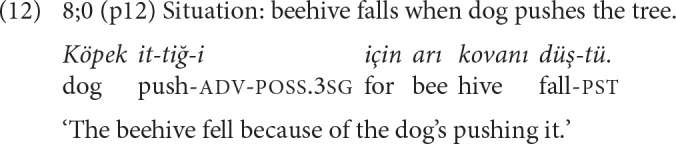



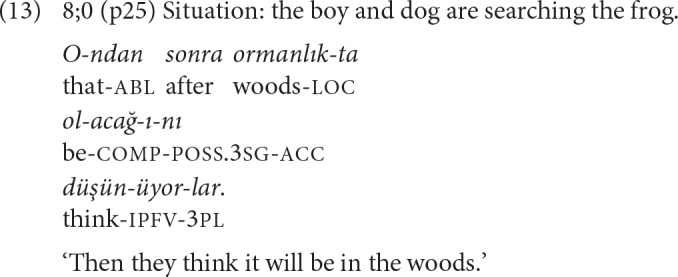


Errors are also observed in the use of 1-NCL complex sentences with these clause types. In example (14a) the subject of the complement clause lacks the genitive suffix and its verb the accusative suffix. In addition, a verb for the adverbial clause implied by the postposition *için* ‘for' is missing. The correct version is given in example (14b).


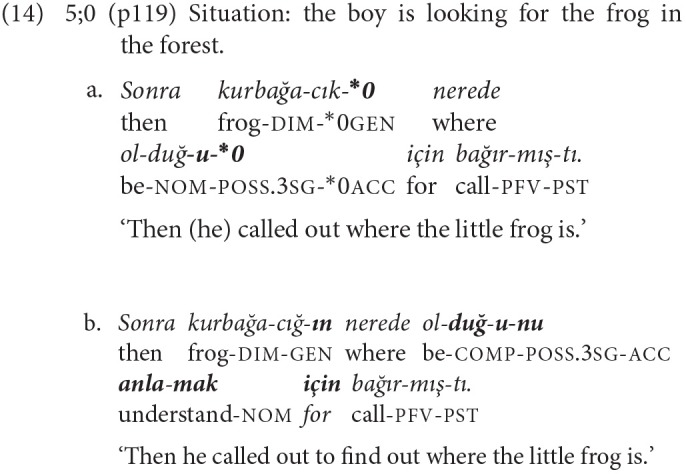


These examples illustrate that morphosyntactic complexity affects young children's production of complex sentences. The errors concerning the use of –*IncE* mentioned above, on the other hand, suggest that it is not just complex morphosyntax that causes problems, but the conceptual coordination of the activities of different actors may also be at issue.

#### 2-NCL and 3-NCL Complex Sentences

In Table 7, we have the distribution by age of 2- and 3-NCL clause chains and other non-finite clause combinations. It is observed that the number of complex sentences composed of converbal clauses only, i.e., chains, is limited for each age group.

**Table 7:** Frequency (and percentage) of clause types in 2- and 3-NCL chains and other non-finite clause combinations by age^*^.

		**Chains**	**Other non-finite clause combinations**				
**Age**	**Total**	**CVB +CVB (+COMP)**	**CVB + ADV (+REL)**	**CVB + COMP**	**CVB+ REL**	**ADV + ADV (+REL)**	**ADV + COMP**
4- years	–	–	–	–	–	–	–
5- years	7	3(+2)	–	2	–	–	–
		(71.43)		(28.57)			
8- years	5	–	1	–	–	2	2
			(20.00)			(40.00)	(40.00)
11- years	16	4	4(+1)	3	1	1(+1)	1
		(25.00)	(31.25)	(18.75)	(6.25)	(12.50)	(6.25)
adults	18	4(+1)	5(+1)	2	2	2	1
		(27.77)	(33.33)	(11.11)	(11.11)	(11.11)	(5.56)

* *The frequencies of 3-NCL clauses are indicated in parenthesis following the + sign*.

4-year-olds do not produce any 2-NCL or 3-NCL complex sentences. Chains with at least two converbal clauses constitute 71.43% of the complex sentences for 5-year-olds, 25.00% for 11-year-olds and 27.77% for adults. 8-year-olds do not produce any two converbal chains. Combinations with at least one converbal clause and an associated adverbial or an embedded complement or relative clause constitute 28.57% of the complex sentences for 5-year-olds, 20% for 8-year-olds, 56.25% for 11-year-olds and 55.55% for adults. Complex sentences composed of adverbial and/or complement or relative clauses are 80% for 8-year-olds but do not exceed 20% for the older participants.

Below, we discuss the predominant patterns of clause chains and other combinations for each age group.

##### 5-year-olds

The first clause chains with more than one converbal clause are observed in the 5-year-old narratives. Example (15) illustrates the repeated use of -*Ip* to relate the actions of the same actor in temporal succession, and (16) the repeated use of -*ken* to relate the activities of different actors as simultaneous.


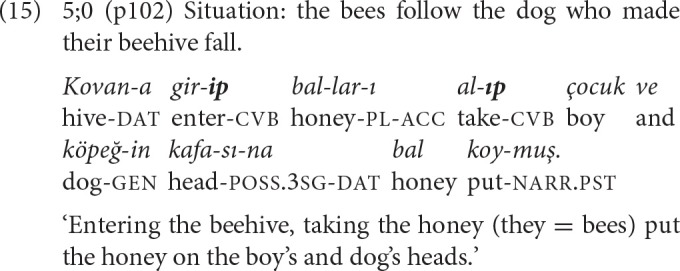



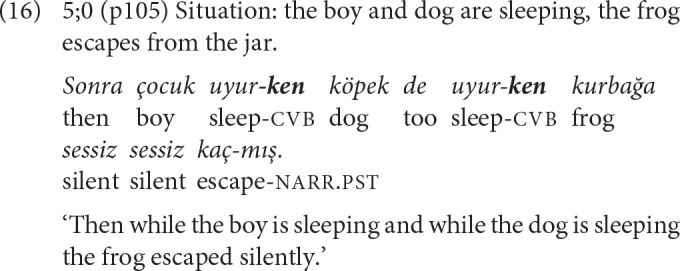


As Table 7 shows, two 5-year-olds also produced 3-NCL complex sentences composed of a chain of two repeated converbal clauses with a complement clause. In the SS chain of example (17), the first -*IncE* clause sets the temporal frame, and the second -*IncE* clause presents the perception of the absence of the frog expressed with an embedded complement clause, as the cause for the event of the main clause.


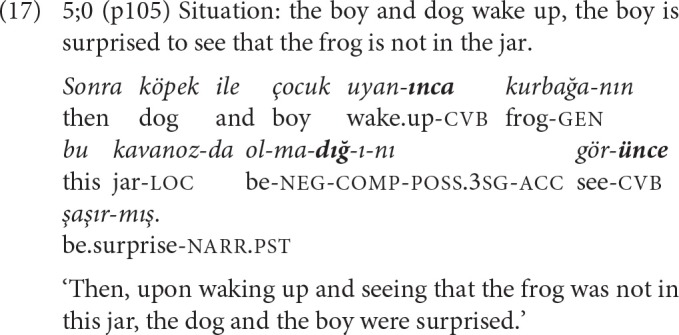


These examples suggest that repeating the same chaining device in successive clauses to relate sequential activities of the same actor or simultaneous activities of different actors may be the easiest strategy for young children, conceptually as well as syntactically. This strategy also ensures chain-internal aspectual continuity, for example, perfective aspect with the repetition of *-Ip* clauses and imperfective aspect with the repetition of *-ken* clauses, and also contributes to chain-internal referential continuity, as discussed in Section on Referential Continuity.

##### 8-year-olds

For 8-year-olds, the main linking strategy is event packaging with adverbial clauses. Only one of the five complex sentences contains a converbal clause combined with an adverbial clause; the rest are combinations of two adverbial or an adverbial and a complement clause (Table 7) and reveal a shift of emphasis from temporal to causal relations. Examples like (18) where a converbal and an adverbial clause present the temporal-causal background, and (19) where two adverbial clauses present the causal basis for the event of the main clause, show that complex sentences with adverbial clauses may also function like chains.


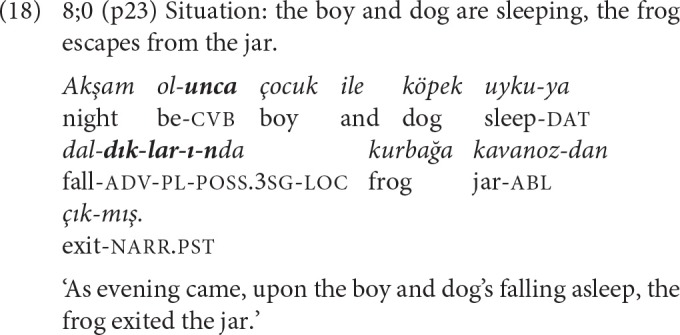



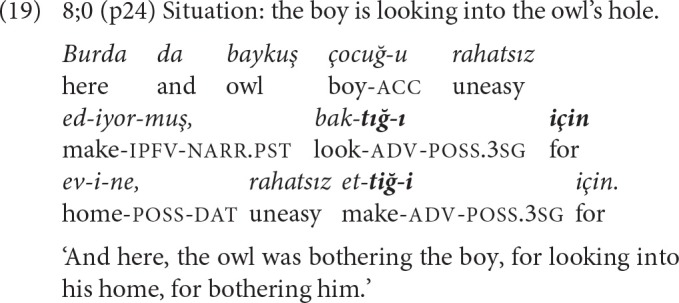


Although 8-year-olds produce complex combinations of converbal, adverbial and complement clauses, some of the examples still display errors such as the omission of the accusative marker on the verb of the complement clause and the agreement marker on the verb of the adverbial clause, similar to the errors of 5-year-olds. Such morphosyntactic errors prevailing at 8 years point to the processing demands of formulating complex language when the task is one of producing organized narrative discourse.

##### 11-year-olds

Three of the four 2-NCL chains found in the narratives of this age group are formed with the repetition of the same converb, similar to the pattern observed for 5-year-olds. In example (20), two SS *-ErEk* clauses are chained to describe the psychological state of the actor and his consequent action from an integrated imperfective perspective, illustrating the conceptually complex discourse function of this form (Slobin, 1995, p. 349).


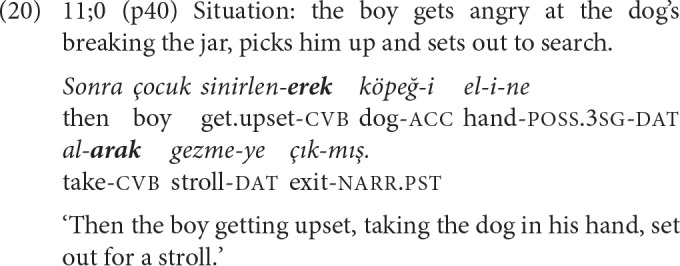


In the other two examples where the same converbal suffix (e.g., *-Ip* and -*Ip*, or -*ken* and -*ken*) is repeated in the chained clauses, either different activities of the same actor, or similar activities of different actors are related. A deviation from this pattern is observed in the use of two different converbs *-ken* and *-Ip*, connecting the ongoing activity of one actor to the temporally overlapping activity of a second one, as in example (21) where both clauses have overt subjects expressed with an NP, and the clause marked with *-Ip* is followed by the main clause with a null pronoun for subject, as signaled by the SS clause.


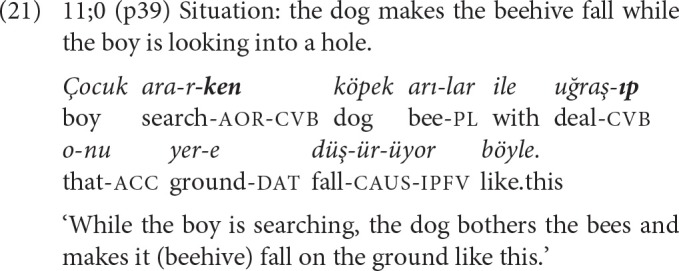


The advance observed in the complex sentences of 11-year-olds is the increased variety of the types of non-finite clauses combined. In a total of eight 2-NCL and one 3-NCL complex sentence, a converbal clause is combined with an adverbial, a complement or a relative clause. In such combinations, a *-ken* or an *-Ip* converbal clause functions to frame events in terms of temporal and/or referential continuity. Example (22) is a 3-NCL complex sentence composed of a converbal, a relative, an adverbial and a main clause used to introduce the plot-initiating event of the story.


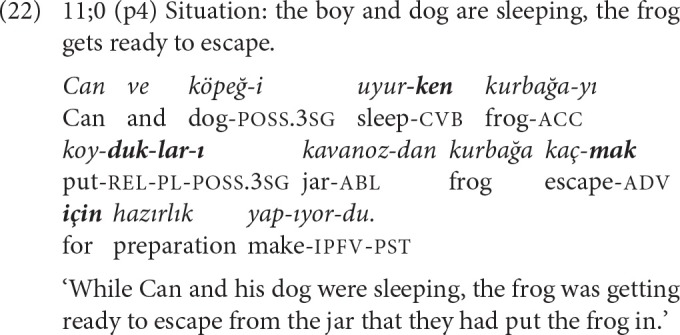


Older children can easily situate events referred to by an *-Ip* or -*ErEk* clause against background events expressed by an *-IncE, -ken* or adverbial clause, and further refer to the mental states of actors with a complement clause embedded in the sequence, thus producing complex sentences that can be regarded as ‘chains with other non-finite clauses.' These advances in children's use of language reflect developments in their ability to coordinate actions and mental states of different actors, as well as in their developing narrative skills for backgrounding, foregrounding, and evaluating events from different perspectives.

Chains showing increased variability in terms of the relations contracted between clauses by use of different converbs are found more frequently in the narratives of the adults. Of the five full chains observed for adults, only one is a combination of -*ken* and -*ken* clauses, while two are -*ken* and -*Ip*, and two are -*dIktEn sonra* and *-Ip* combinations.

### Continuity in Clause Chains and Other Non-finite Clause Combinations

As Givón puts it “… coherent discourse tends to maintain, over a span of several clauses, the same topical referent, the same or contiguous time, the same or contiguous location, and sequential action” (2017a, p. 29; also Watanabe, 1994). Following this idea, our second research question asked whether children's 2- and 3-NCL complex sentences showed a developmental pattern in terms of the type of discourse continuity contracted. Below, we present the distribution of chains and clause combinations in terms of the aspectual-temporal and referential continuities they display.

#### Aspectual-Temporal Continuity

We define aspectual-temporal continuity internal to a complex sentence (for clause chains or clause combinations separately) as the use of forms that encode the same aspectual distinctions in successive clauses (e.g., all encoding perfective or all encoding imperfective aspect). The patterns observed across 2- and 3-NCL chains and other non-finite clause combinations are summarized in Table 8. 1-NCL chains were not included in this analysis because the anchor tense marked on the main verbs of each narrative did not show any variation.

**Table 8:** Frequency (and percentage) of 2-NCL and 3-NCL complex sentences in terms of type of aspect by age.

		**Chains**			**Other combinations**		
	**Total**	**Same aspect**	**Different aspect**	**Aspect and modality**	**Same Aspect**	**Different Aspect**	**Aspect and modality**
4- years	–	–	–	–	–	–	–
5- years	7	4 (57.14)	1 (14.29)	–	–	2 (28.57)	–
8- years	5	–	–	–	3 (60.00)	1 (20.00)	1 (20.00)
11- years	16	3 (18.75)	1 (6.25)	–	2 (12.5)	7 (43.75)	3 (18.75)
adults	18	3 (16.67)	2 (11.11)	–	4 (22.22)	7 (38.89)	2 (11.11)

For 5-year-olds, complex sentences with chains display a high percentage of same aspect clauses (57.14%) whereas the complex sentences with other non-finite clause combinations display different aspectual specifications (28.57%). Eight-year-olds do not produce any clause chains, but their other non-finite clause combinations also show a preference for the same (60%) in comparison to different (20%) aspectual marking. Eleven-year-olds and adults, on the other hand, produce more complex sentences with other non-finite clauses (75% and 72%, respectively) than chains, and majority of these express different aspectual combinations. However, the dominant pattern across the clauses of their chains is same aspectual marking (3 out of 4, for 11-year-olds and 3 out of 5 for adults). These distributions in Table 8 indicate that younger children install continuity at the sentential level by using clause chaining devices that present events from the same aspectual-temporal framework, as in examples (15) and (16). Older children and adults, on the other hand, tend to use varied combinations of converbal, adverbial, complement and relative clauses for expressing relations that hold beyond the sequential clausal level and from different perspectives as in example (22).

#### Referential Continuity

Referential continuity was evaluated for chains only. We define referential continuity internal to a chain in terms of the identity of subjects expressed by an NP, pronoun or a null pronoun, and cross-sentence-boundary referential continuity (XSR) in terms of the identity of subjects anaphorically referred to by a null pronoun or pronoun to an NP in one of the previous clauses external to the chain.

Table 9 summarizes the frequency of types of referential continuity by age. The category ‘DS' refers to chains that link the activities of different actors, and therefore lack referential continuity. The category ‘SS' refers to chains that link different activities of the same actor and therefore establishes refrential continuity. The third type of referential combination, observed in case of 2- and 3-NCL chains, is ‘2SS-1DS,' where two of the actors are the same and one is different.

**Table 9:** Frequency (and percentage) of DS, SS and 2SS-1DS combinations in 1-, 2- and 3-NCL chains and cross-sentence-boundary referential continuity (XSR) by age.

**Age**	**Total**	**DS chains**	**SS chains**	**2SS-1DS chains**	**Total XSR**	**All DS XSR**	**All SS XSR**	**2SS-1DS XSR**
4- years	23	9 (39.13)	14 (60.87)	–	15 (65.22)	6 (40.00)	9 (60.00)	–
5- years	42	16 (38.10)	25 (59.52)	1 (2.38)	25 (59.52)	7 (28.00)	18 (72.00)	–
8- years	34	10 (29.41)	24 (70.59)	–	16 (47.06)	4 (25.00)	12 (75.00)	–
11- years	57	17 (29.82)	39 (68.42)	1 (1.75)	21 (36.84)	6 (28.57)	15 (71.43)	–
adults	32	9 (28.12)	22 (69.75)	1 (3.13)	16 (50.00)	5 (31.25)	10 (62.50)	1 (6.25)

It is observed in Table 9 that among 1-, 2-, and 3-NCL chains, the proportion of SS chains is higher (ranging between 60.87% and 70.59%) than that of DS chains (ranging between 28.12% and 39.13%) at all ages. The proportion of SS chains which is around 60% for 4- and 5-year-olds is somewhat higher in the narratives of the older age groups (around 70%). Furthermore, the proportion of chains with cross-sentence-boundary anaphoric reference is higher for SS than for DS chains (ranging between 60% and 75%) at all ages. These distributions indicate that by the age of 4 children are using chaining devices effectively, achieving referential continuity intra-sententially, and also beyond sentence boundaries, thus maintaining continuity with a previous discourse segment,

In example (23) from an 11-year-old, two *-Ip* clauses relate the activities of the same actor from a perfective-sequential perspective and contract both aspectual-temporal and referential continuity. Furthermore, the SS *-Ip* suffix makes anaphoric reference across the sentence boundary to an NP that went before, as well as signaling that the subject of the upcoming clause is the same.


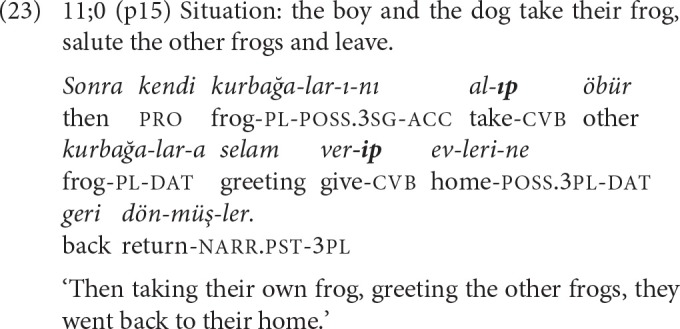


In summary, evaluated from the perspective of continuity, the acquisition data suggest that Turkish children's earliest non-finite clause combinations and chains are built on aspectual and referential continuity, then differentiate to include temporal-causal perspectives, and coordination of subjects.

## Discussion and Conclusion

In the present study, we investigated the development of complex sentences with non-finite clauses of varying degrees of dependency and embedding in the narratives of Turkish-speaking 4- to 11-year-olds and adults. Our first research question concerned developments in clause chaining with converbal clauses that are dependent but not embedded, as well as developments in clause combining with complement, relative and adverbial clauses that involve both dependency and embedding.

Our results show an early start and a gradual change across age groups. In terms of frequency, a clear increase in 1-, 2- and 3-NCL complex sentences is observed by age, the performance of 11-year-olds approximating that of adults.

In terms of length, 4-year-olds produce maximally 1-NCL sentences, while older children and adults produce 2-NCL and occasionally 3-NCL sentences. Since the number of clauses a chain can potentially include is far above three observed in the present data, our findings need some explanation. First, it may be that the elicitation material that provides two or three events at a time in the pictures of the story book, thus the content to be told, could have limited the length of the chains. Second, longer chains, even 2- and 3-NCL clause ones might have been scarce because narrators, particularly older ones, present event packages where they combine converbal with adverbial, complement, and relative clauses. Third, this may be the nature of clause chaining in Turkish, where length of chains is rather limited to 3 or 4 converbal clauses.

In terms of the variety of clause types combined into a complex sentence, converbal and adverbial clauses are recorded from age 4 on, complement clauses appear at 5, and relative clauses are found only in the narratives of 11-year-olds and adults. The variety of clause types combined in a complex sentence also increases with age. The 2- and 3-NCL complex sentences of 5-year-olds consist mainly of combinations of converbal clauses and very few complement clauses, while those of 8-year-olds comprise adverbial and complement clauses, and those of 11-year olds and adults, combinations of converbal, complement, adverbial and relative clauses.

Among converbal clauses functional in clause chains, those with the SS -*Ip* suffix for sequencing emerges first and is the most frequent at all ages. Next are the SS/DS converbs, *-IncE* for relating events in temporal-causal succession, *-ken* for representing simultaneity, and -*DIktEn sonra* for temporal ordering of events. The SS converb -*ErEk*, which presents events from a temporally integrated perspective, is observed mainly in the stories of 11-year-olds and adults. The frequency of SS chains is higher than that of DS chains across all age groups. Adverbial clauses provide the temporal-causal circumstances for plot-advancing events in 8- and 11-year-olds' narratives, and complement clauses are used to make mental state attributions by 11-year-olds and adults. These developments are not error-free, however. Violation of the SS vs. DS subject requirements of converbs, and failure to provide the person/number and/or genitive suffixes for subject reference of adverbial and complement clauses, or the accusative case on complements reveal the semantic and morphosyntactic complexity of clause combining structures in Turkish.

Our second research question examined whether types of clauses that enter clause chains or clause combinations in complex sentences contribute to discourse continuity in children's narratives. We observed that younger children present events from the same aspectual temporal framework using converbal clauses in chains, while older children and adults present events from varied perspectives, using other non-finite clauses in clause combinations. As for referential continuity, we observed that children use SS chains more frequently than DS chains and make use of null pronouns across sentence boundaries from age 4 onwards. These patterns indicate that Turkish-speaking children are quite competent in establishing both sentence-internal and cross-sentence-boundary referential continuity during the preschool years.

Evaluated from the syntactic point of view, the observed developmental trajectory indicates that converbs are easier and earlier accessed by children than adverbial, complement and relative clauses. This is not surprising since the morphosyntactic mechanisms involved in the construction of this latter group (nominalization, person/number marking on the embedded verb and/or case marking on its subject) are more complex than those involved in converbal clauses functional in clause chaining. An interesting question raised by these findings concerns the usage of direct speech reports and clauses conjoined with *ki* and *diye*, both embedded but not dependent. Although not included in the present analysis, the overview of sentence types used in the narratives (Table 2) showed that direct speech reports constitute 48% of the complex sentences used by 4-year-olds, showing a decrease with age to 2.5% at 11 years, and clauses conjoined with *ki* and *diye*, though 19% in the narratives of 8-year-olds, decrease to 3% and 6% in those of 11-year-olds and adults. The high frequencies of these structures at younger ages, particularly those of direct speech reports, shows that embedding *per se*, when dependency is not involved, may not pose difficulty, but that it is the combination of embedding and dependency that Turkish children find as a source of complexity in acquisition. A systematic comparison of structures with different combinations of these features remains to be a problem for future research.

Our findings concerning the functional development of chaining devices in children's narratives confirm the results of earlier studies on Turkish (Aksu-Koç, 1994; Slobin, 1995; Aarsen, 1996; Çapan, 2013; Rehbein and Herkenrath, 2015) as well as the cross-linguistic trends regarding event packaging reported by Berman and Slobin (1994a). Putting them together, we interpret the observed trajectory in language development in terms of two developmental shifts in children's cognitive abilities.

The first shift is between ages 4 and 5, as reflected in the emergence of 2- and 3-NCL complex sentences in the narratives of 5-year-olds compared to 4-year-olds. One explanation for this break may be in terms of the richness of their verb repertoire, i.e., their knowledge of the syntactic and semantic properties of verbs that can figure in combinations of other non-finite clauses (e.g., complement clauses). As was observed, the verb diversity of 4-year-olds is restricted as compared to that of older children, which could have affected their skill in clause combining. A second explanation for the fact that 4-year-olds produce 1-NCL sentences but not longer ones may be in terms of processing constraints rather than the syntax or semantics of these devices. Constructing complex sentences, whether by clause chaining or combining, requires planning, holding the subordinate units in working memory and inhibiting irrelevant information, all subskills of executive functions. In narrative discourse in particular, remembering the subject of a previous clause, and evaluating whether it is same as, or different from the one(s) in the current and upcoming clauses is important for referential continuity and for shifting perspectives (Drijbooms et al., 2017). The effects of these processing constraints are likely to be somewhat reduced between the ages of 4 and 5, the age bracket when executive functions show a significant development (Friend and Bates, 2014).

The second qualitative shift is observed between ages 8 and 11, as revealed by the higher frequency of use of complement clauses which refer to the mental sates of the actors, and relative clauses which qualify referents in terms of their context-relevant properties, in the narratives of 11-year-olds compared to those of 8-year-olds. These changes in children's use of expressive means are observed along with changes in the conceptual content of their narratives. We suggest that such differences are due to advances in children's theory-of-mind capacities which allow them to integrate the perspectives of temporally and spatially displaced story characters into the event structure of the narrative through the use of the syntactic means they command. These relationships between complex sentence construction, executive function and ToM deserve future research in different discourse genres, using larger samples.

The need for an extended data base coming from larger samples and different discourse genres brings us to a consideration of the limitations of the present study due to the nature of the stimulus and the elicitation procedure used. As discussed above, relating events in terms of a coherent narrative and expressing them in a sequence of clauses present conceptual and processing demands. The 24-page wordless storybook was, therefore, kept in front of the narrators, with the successive pages of the book opened in tempo with the telling. The content of the pictures, with two protagonists engaged in two different activities, can be said to have set the boundaries for the content of the utterances, leading to the production of a limited number of chains of greater length. Future research should, therefore, use procedures where spontaneously self-generated narratives in everyday contexts are elicited.

In conclusion, our findings show a developmental trajectory where chaining devices, i.e., converbs, are the earliest non-finite clause linking structures children use, and that the pace of development is different but slower for other non-finite clause types. They demonstrate that the frequency, diversity and length of clause chains and clause combinations with other non-finite structures increase with age. Children's earliest clause chains are built on referential and aspectual-temporal, then on temporal-causal continuity. Referential continuity across sentence boundaries indicates that the scope of cohesive ties installed by referential means covers larger segments of discourse than individual clause chains. And most significantly, acquisition of these types of complex sentences rests not only on linguistic competence but also on conceptual development and processing skills.

## Data Availability Statement

The data for this study are available on request to the corresponding author.

## Ethical Statement

All procedures performed in studies involving human participants were in accordance with the ethical standards of the institutional and/or national research committee and with the 1964 Helsinki declaration and its later amendments or comparable ethical standards.

## Author Contributions

HÖ-B designed the study, collected data, conducted data analyses, and collaborated on the writing of the paper. AA-K designed the study, conducted data analyses, and wrote the paper.

### Conflict of Interest

The authors declare that the research was conducted in the absence of any commercial or financial relationships that could be construed as a potential conflict of interest.

## Footnotes

^1^Affixes alternate according to the rules of vowel harmony, which operate in terms of the high/low, front/back and rounded/unrounded phonological contrasts. Consonant assimilation and other regular morphophonological processes also apply. Alternating vowels and consonants are represented by uppercase characters.

^2^The examples are taken from the adult narrators of the present study. Age, participant identification number and situational information are given in the first line of each example. AD stands for ‘adult'.

^4^Slobin (1995, p. 349) reports from Von Gabain (1941) that some version of -*Ip* is the oldest and most widespread clause chaining form in the Turkic languages. In Foley and van Valin's (1984) framework, converbs would be included under co-subordination (Erguvanlı Taylan, 1988).

^6^Infinitival complement clauses with -*mEk* followed by an aspectual or modal verb as in *Köpek ar**ılardan kaçmak istiyordu* ‘The dog wanted to run away from the bees' were not included since they do not link two events but focus on a phase of a single situation.

^7^Non-finite clauses that are dependent but not embedded have been classified as co-subordination by Foley and van Valin (1984), while clauses that are embedded but not dependent are not discussed in their framework. This latter type is possible with a few constructions in Turkish, involving the verb *san-* ‘presume' or the conjunctions *diye* and *ki* (Erguvanlı Taylan, 1988, p.339; Kornfilt, 1997, p.46).

## Abbreviations

1: First person
2: Second person
3: Third person
ABL: Ablative
ACC: Accusative
ADV: Adverbial clause
AOR: Aorist
CAUS: Causative
CM: Compound marker
COMP: Complement clause
CVB: Converb
DAT: Dative
DIM: Dimunitive
FUT: Future
GEN: Genitive
IPFV: Imperfective
LOC: Locative
NARR.PST: Narrative past
NEG: Negative
NOM: Nominalizer
PL: Plural
POSS: Possessive
PFV: Perfective
PSB: Possibility
PST: Past/Perfective
REFL: Reflexive
REL: Relative clause
SG: Singular.
